# Suicidal Behavior in US Army Special Operations Forces

**DOI:** 10.1001/jamanetworkopen.2025.27395

**Published:** 2025-08-15

**Authors:** James A. Naifeh, Robert J. Ursano, Rachel Shor, Holly BH. Mash, Pablo A. Aliaga, Carol S. Fullerton, Tzu-Cheg Kao, Nancy A. Sampson, Ronald C. Kessler, Murray B. Stein

**Affiliations:** 1 Center for the Study of Traumatic Stress, Department of Psychiatry, Uniformed Services University of the Health Sciences, Bethesda, Maryland; 2 Henry M. Jackson Foundation for the Advancement of Military Medicine, Bethesda, Maryland; 3 Department of Preventive Medicine and Biostatistics, Uniformed Services University of the Health Sciences, Bethesda, Maryland; 4 Department of Health Care Policy, Harvard Medical School, Boston, Massachusetts; 5 Department of Psychiatry, University of California, San Diego, La Jolla; 6 Herbert Wertheim School of Public Health, University of California, San Diego, La Jolla; 7 VA San Diego Healthcare System, San Diego, California

## Abstract

**Importance:**

Research on suicide risk among the US Army’s elite special operations forces (SOF) has been extremely limited.

**Objective:**

To examine suicidal behaviors during the Iraq and Afghanistan war in the 2 major SOF elements: operators (ie, elite soldiers trained in unconventional warfare), and support personnel (ie, soldiers who assist operators and SOF missions).

**Design, Setting, and Participants:**

This retrospective cohort study of administrative data identified all operator, support, and other regular Army enlisted person-months from 2004 through 2012 with a first nonfatal suicide attempt and an equal-probability control sample. Separate case-control samples were drawn to examine suicide death and suicide ideation. Analyses were carried out from July 8, 2024, to May 27, 2025.

**Exposure:**

Serving as an SOF operator or support soldier.

**Main Outcomes and Measures:**

Suicide ideation and nonfatal suicide attempt were identified using *International Classification of Diseases, Ninth Revision, Clinical Modification *codes and Department of Defense Suicide Event Report records. Suicide death were identified using the Armed Forces Medical Examiner Tracking System. Sociodemographic, Army career, and mental health variables were constructed from administrative personnel and medical records.

**Results:**

The total weighted sample of regular Army enlisted soldiers was 48 103 189 person-months (41 717 105 male [86.7%]; mean [SD] age, 28.8 [8.6] years; 32 263 202 younger than aged 30 years [67.1%]). Operator suicide attempt crude rates were 70% lower than those of SOF support personnel (rate ratio [RR], 0.3; 95% CI, 0.2-0.3) and 90% lower than those of the total regular force (RR, 0.1; 95% CI, 0.1-0.2). Operator suicide ideation crude rates were 70% lower than rates for SOF support personnel (RR, 0.3; 95% CI, 0.2-0.3) and 80% lower than those of the total regular enlisted force (RR, 0.2; 95% CI, 0.1-0.2), whereas suicide death rates did not differ. The ratio of suicide attempts to suicide deaths was 2.1 (95% CI, 1.9-2.3) for operators, 7.6 (95% CI, 7.5-7.7) for support personnel, and 15.0 (95% CI, 15.0-15.0) for the total regular force. Adjusting for sociodemographic and Army career differences attenuated but did not eliminate operators’ lower risk of suicide attempt and suicide ideation. In multivariable analyses, only 3 of 9 risk factors were associated with suicide attempt among both operators and support personnel: lower rank, past-year demotion, and mental health diagnosis during service. Odds were higher for female operators (OR, 7.4; 95% CI, 3.2-17.1). Sex was not associated with attempts among support personnel.

**Conclusions and Relevance:**

In this study of US regular Army enlisted soldiers during the Iraq and Afghanistan wars, SOF operators were less likely than other soldiers to engage in suicidal behavior, but it was more likely to be fatal when they did. Larger studies are needed to understand suicide risk among female operators.

## Key Points

**Question:**

Does suicide risk differ for US Army special operations forces (SOF) operators (elite soldiers trained in unconventional warfare), SOF support personnel (soldiers who assist operators and SOF missions), and all other soldiers?

**Findings:**

In this cohort study with 48 103 189 person-months (PM) from regular enlisted soldiers, including 980 406 PM from SOF operators and 1 780 514 PM from SOF support soldiers, SOF operators had lower rates of suicide ideation and suicide attempt than other soldiers but similar rates of suicide death, even after adjusting for sociodemographic and career differences.

**Meaning:**

These findings suggest that although operators are less likely than other soldiers to engage in suicidal behavior, it is more likely to be fatal when they do.

## Teaser

This cohort study examines risk of suicidal behaviors during the Iraq and Afghanistan war among US Army special operations forces and regular enlisted soldiers.

## Introduction

US Army Special Operations Forces (SOF) played a critical role in the wars in Afghanistan and Iraq from the earliest days following the terrorist attacks of September 11, 2001.^1^ Over the 2 decades of war that followed, there were substantial increases in the Army-wide rates of suicide death and nonfatal suicide attempt, which remain elevated today.^2,3,4^ Although suicide within the SOF community has been a subject of significant concern,^5,6^ few studies have been done.

Operators and support personnel are the 2 major elements of SOF. Operators are elite soldiers who undergo a rigorous selection process, including mental health screening, and advanced training for unconventional warfare. They often maintain a high operational tempo, meaning a heightened frequency and intensity of deployments, training, and other military activities. Support personnel, sometimes referred to as enablers, are soldiers who serve within SOF units and support operators and SOF missions but do not go through the same selection and training as operators. The few published studies of SOF mental health typically focus exclusively on operators or do not differentiate operators from support personnel. Previous findings indicate that operators have lower risk of mental and behavioral health problems than most other soldiers, including those in regular force combat roles, such as infantry.^7,8,9,10^ One study found that operators and SOF support personnel do not differ in odds of posttraumatic stress disorder or depression after controlling for demographic differences, but operators had higher odds of alcohol problems.^9^ A representative study of suicidal behavior among the subgroup of operators known as Green Berets found that operators had substantially lower risk of documented nonfatal suicide attempt than other soldiers with a direct combat occupation.^7^ Importantly, no studies to date have examined suicide ideation, nonfatal suicide attempt, or suicide death in a fully representative sample of regular Army SOF.

Using Army and Department of Defense (DOD) administrative data from the Army Study to Assess Risk and Resilience in Servicemembers (Army STARRS),^11^ we examine rates of suicide ideation, nonfatal suicide attempt, and suicide death among regular Army enlisted operators and SOF support personnel from 2004 through 2012, a period encompassing the height of the wars in Iraq and Afghanistan, and compare them with rates among the total regular Army enlisted force. We also consider whether differing rates of suicidal behavior between operators and other soldiers are attributable to differences in basic sociodemographic and Army career characteristics which are both known to be associated with suicide-related outcomes and likely to differ between operators and other soldiers. Lastly, we examine risk factors for suicide attempt within SOF, and whether associations of those risk factors differ for operators vs support personnel.

## Methods

### Sample

The Army STARRS Historical Administrative Data Study (HADS) is a longitudinal, retrospective cohort study that integrates 40 Army and DOD administrative data systems, including every system that documents suicidal events (eTable 1 in Supplement 1).^12^ Within the HADS are individual-level person-month records for all regular Army enlisted soldiers who were on active duty at some point from January 1, 2004, through December 31, 2012 (1 070 885 soldiers). The current study focused on regular Army enlisted soldiers, excluding officers, Army National Guard, and Army Reserve, which differ from regular Army enlisted soldiers in military career experiences, sociodemographic characteristics, and risk for suicidal behavior.^13,14,15^ Additionally, National Guard and Reserve soldiers typically do not have access to military health care during periods when they are deactivated from federal service, substantially limiting the capture of suicide ideation and suicide attempt in those populations. We selected 3 independent, 1:20 case-control samples, 1 for each suicide-related outcome (nonfatal suicide attempt, suicide death, and suicide ideation). The analytic sample for nonfatal suicide attempt included all 14 547 person-months in which a first suicide attempt occurred from 2004 through 2012 and an equal-probability sample of 149 166 control person-months in which a documented suicide attempt did not occur (person-months in which a soldier died were excluded). Data were analyzed using a discrete-time survival framework with person-month as the unit of analysis.^16,17^ Control person-months were weighted based on the inverse probability of selection. Additional details can be found in Supplement 1.

An identical process was used to create the analytic samples for suicide death (972 cases; 15 630 control person-months) and suicide ideation (25 584 cases; 278 708 control person-months), the latter of which was not administratively documented until 2006 (ie, a 2006 through 2012 study period). The Army STARRS HADS was approved by the institutional review board of the University of Michigan Institute for Social Research, with secondary review and approval by the Uniformed Services University, University of California–San Diego, and Harvard Medical School. This study followed the Strengthening the Reporting of Observational Studies in Epidemiology (STROBE) reporting guideline. These institutional review boards determined that consent was not required because secondary analysis of the deidentified data in the HADS did not constitute human participant research.

#### Identifying SOF

Special operations units were identified based on unit identification codes (UICs), which are alphanumeric codes used to uniquely identify and track units, assign personnel, and manage logistics. Person-months were identified in which either the soldier’s assigned unit or duty unit had a special operations UIC. Regular Army enlisted soldiers who had ever served within those units during the study period were separated into operators (eTable 2 in Supplement 1) and support personnel. Each person-month was coded to indicate whether the soldier was currently or not currently serving in a SOF unit.

### Measures

#### Suicidal Thoughts and Behaviors

Suicide deaths, which are investigated by the Army’s criminal investigation division, were identified using the Armed Forces Medical Examiner Tracking System. Nonfatal suicide attempts, which are diagnosed based on clinician judgment, were identified using DoD Suicide Event Report (DoDSER) records^18^ and *International Classification of Diseases, Ninth Revision, Clinical Modification *(*ICD-9-CM*) codes E950-E958 from multiple administrative medical systems. Administratively documented suicide ideation, also diagnosed based on clinician judgement, was identified using DoDSER records and *ICD-9-CM* code V62.84 (eTable 1 in Supplement 1).

#### Sociodemographic and Army Career Characteristics

Administrative personnel records were used to derive sociodemographic variables (sex, age, marital status, and race-ethnicity dichotomized as White non-Hispanic vs other [ie, American Indian or Alaska Native, Asian, Black, Hispanic, and Native Hawaiian or Other Pacific Islander] owing to small cell sizes for some outcomes; see eTable 3 in Supplement 1 for original database categories]) and Army career variables (military occupational specialty [SOF operator, SOF support, and all other enlisted soldiers], rank, deployment status, and past-year demotion).

#### Mental Health Diagnosis

*ICD-9-CM* codes were used to create an indicator variable for previous in-service mental health diagnosis (including V-codes for stressors, adversities, and marital problems) (eTable 4 in Supplement 1).

### Missing Values

During creation of the HADS, some item-level administrative data were missing for particular person-months. These data were typically recovered by cross-checking other data systems or other months in the same soldier’s records. The few missing values that could not be recovered through cross-checking were imputed based on subgroup modes.

### Statistical Analysis

Analyses were conducted using SAS version 9.4 (SAS Institute).^19^ The weighted samples were used in all analyses. Crude rates of suicide ideation, nonfatal suicide attempt, and suicide death were calculated for operators, support personnel, and the total regular Army enlisted population, then compared with risk ratios (RRs). We then used the coefficients from multivariable logistic regression models that adjusted for group differences in sociodemographic and Army career variables (sex, age, race-ethnicity, marital status, rank, deployment status) to generate standardized risk estimates (SREs)^20^ for suicide ideation, suicide attempt, and suicide death (per 100 000 person-years) among operators, support personnel, and all other regular enlisted soldiers. Logistic regression analyses, conducted separately among operators and support personnel, examined associations of sociodemographic, Army career, and mental health diagnosis variables with suicide attempt in a given person-month, with all risk factors other than sex and race-ethnicity treated as time-varying covariates. Two-way interactions between SOF element (operator vs support personnel) and the other explanatory variables were examined in separate multivariable models. The same approach was used to examine risk factors for suicide ideation. Logistic regression coefficients were exponentiated to obtain odds ratios (ORs) and 95% CIs. All logistic regression models included a dummy variable for calendar month and year to control for secular trends.

## Results

### Sample Characteristics

The total suicide attempt case-control sample for operators included 993 308 weighted person-months with the following distribution of soldier characteristics: 980 406 male (98.7%), 802 450 White Non-Hispanic (80.8%), 453 623 age 30 years or older (54.3%), 637 378 currently married (64.2%), 878 202 rank E5 or higher (88.4%), 833 718 currently or previously deployed (83.9%), 9677 demoted in the past year (1.0%), and 307 912 with a previous mental health diagnosis (31.0%). From 2004 through 2012, 45 operators had a nonfatal suicide attempt (Table 1) and 21 died by suicide (eTable 5 in Supplement 1). From 2006 through 2012, 97 operators had administratively documented suicide ideation (eTable 6 in Supplement 1).

**Table 1.** Distribution of Sample Characteristics by Nonfatal Suicide Attempt Among Special Operations Forces (SOF) Operators, SOF Support, and the Total Regular Army Enlisted Force, 2004 Through 2012^a^

Characteristic	Person-months, No. (%)					
	SOF				All regular enlisted soldiers	
	Operators		Support			
	Suicide attempt cases	Total population^b^	Suicide attempt cases	Total population^b^	Suicide attempt cases	Total population^b^
Sex						
Male	38 (84.4)	980 406 (98.7)	313 (93.2)	1 780 514 (95.5)	11 010 (75.7)	41 717 105 (86.7)
Female	7 (15.6)	12 902 (1.3)	23 (6.8)	83 198 (4.5)	3537 (24.3)	6 386 084 (13.3)
Age, y						
≤24	16 (35.6)	164 109 (16.5)	212 (63.1)	754 589 (40.5)	9580 (65.9)	20 450 621 (42.5)
25-29	14 (31.1)	289 514 (29.1)	73 (21.7)	546 190 (29.3)	3032 (20.8)	11 812 581 (24.6)
30-34	9 (20.0)	226 967 (22.8)	30 (8.9)	277 280 (14.9)	1094 (7.5)	6 975 214 (14.5)
≥35	6 (13.3)	312 718 (31.5)	21 (6.3)	285 653 (15.3)	841 (5.8)	8 864 773 (18.4)
Race or ethnicity						
White non-Hispanic	38 (84.4)	802 450 (80.8)	258 (76.8)	1 258 843 (67.5)	10 021 (68.9)	28 713 105 (59.7)
Other^c^	7 (15.6)	190 858 (19.2)	78 (23.2)	604 869 (32.5)	4526 (31.1)	19 390 085 (40.3)
Marital status						
Not currently married	19 (42.2)	355 930 (35.8)	161 (47.9)	874 142 (46.9)	8172 (56.2)	22 770 052 (47.3)
Currently married	26 (57.8)	637 378 (64.2)	175 (52.1)	989 570 (53.1)	6375 (43.8)	25 333 137 (52.7)
Rank						
E1-E4	16 (35.6)	115 107 (11.6)	240 (71.4)	880 669 (47.3)	12 155 (83.5)	26 690 671 (55.5)
E5-E6	18 (40.0)	411 057 (41.4)	88 (26.2)	738 668 (39.6)	2150 (14.8)	15 512 015 (32.2)
E7-E8	11 (22.4)	467 145 (47.0)	8 (2.4)	244 375 (13.1)	242 (1.7)	5 900 503 (12.3)
Deployment status						
Currently or previously deployed	35 (77.8)	833 718 (83.9)	202 (60.1)	1 393 865 (74.8)	6501 (44.7)	29 655 335 (61.6)
Never deployed	10 (22.2)	159 590 (16.1)	134 (39.9)	469 847 (25.2)	8046 (55.3)	18 247 853 (37.9)
Demoted in the past year						
Yes	5 (11.1)	9677 (1.0)	42 (12.5)	42 274 (2.3)	1712 (11.8)	1 525 941 (3.2)
No^d^	40 (88.9)	983 632 (99.0)	294 (87.5)	1 821 438 (97.7)	12 835 (88.2)	46 577 248 (96.8)
Mental health diagnosis^e^						
Yes	36 (80.0)	307 912 (31.0)	234 (69.6)	588 584 (31.6)	9701 (66.7)	17 437 748 (36.3)
No	9 (20.0)	685 396 (69.0)	102 (30.4)	1 275 128 (68.4)	4846 (33.3)	30 665 441 (63.7)
Total	45 (100.0)	993 308 (100.0)	336 (100.0)	1 863 712 (100.0)	14 547 (100.0)	48 103 189 (100.0)

^a^ This sample of regular Army enlisted soldiers, which includes all person-months with a first suicide attempt (n = 14 547 cases) and an equal-probability sample of controls person-months (unweighted n = 149 166 controls), is a subset of all soldiers in the 2004 through 2012 Army STARRS Historical Administrative Data Study (HADS). Control person-months were assigned a weight of 322.4 to adjust for undersampling.

^b^ Total population includes both cases (ie, person-months with a first suicide attempt) and weighted control person-months.

^c^ The Other race and ethnicity category includes: American Indian or Alaska Native, Asian, Black, Hispanic, and Native Hawaiian or Other Pacific Islander. eTable 3 in Supplement 1 contains a complete list of the original database categories.

^d^ Includes those who have never been demoted as well as those who were demoted more than a year ago.

^e^ Administratively documented mental health diagnosis.

The total suicide attempt case-control sample for SOF support personnel included 1 863 712 weighted person-months with the following distribution of soldier characteristics: 1 780 514 male (95.5%), 1 258 843 White Non-Hispanic (67.5%), 1 300 779 age 29 years or younger (69.8%), 989 570 currently married (53.1%), 1 619 337 rank E6 or lower (86.9%), 1 393 865 currently or previously deployed (74.8%), 42 274 demoted in the past year (2.3%), and 588 584 SOF with a previous mental health diagnosis (31.6%). From 2004 through 2012, 336 support personnel had a nonfatal suicide attempt (Table 1) and 44 died by suicide (eTable 5 in Supplement 1). From 2006 through 2012, 568 support personnel had documented suicide ideation (eTable 6 in Supplement 1). Sample characteristics for the total regular Army enlisted population are presented in Table 1 and eTable 5 and eTable 6 in Supplement 1.

### Rates of Suicidal Behavior

Among soldiers who were ever operators, mean (SD) annual rates of suicide ideation, suicide attempt, and suicide death were 142.1 (69.1), 54.4 (26.3), and 26.4 (25.6) per 100 000, respectively. The mean (SD) annual rates of suicide ideation, suicide attempt, and suicide death were 423.7 (87.2), 216.3 (45.3), and 27.9 (12.0) per 100 000 for SOF support personnel and 798.5 (205.8), 362.9 (68.2), and 24.0 (7.4) per 100 000 for the total regular enlisted force (Figure). The suicide death rate for operators did not differ from that of support personnel (rate ratio [RR], 1.0; 95% CI, 0.6-1.6) or the total regular enlisted force (RR, 1.1; 95% CI, 0.7-1.7), nor did support personnel differ from the regular force (RR, 1.2; 95% CI, 0.9-1.6). In contrast, the suicide attempt rate among operators was 70% lower than that of support personnel (RR, 0.3; 95% CI, 0.2-0.3) and 90% lower than that of the total regular force (RR, 0.1; 95% CI, 0.1-0.2), and the support personnel rate was 40% lower than that of the regular force (RR, 0.6; 95% CI, 0.5-0.7). The pattern for suicide ideation was similar to that of attempts, with operators having a rate 70% lower than support personnel (RR, 0.3; 95% CI, 0.3-0.4) and 80% lower than that of the total regular force (RR, 0.2; 95% CI, 0.1-0.2), and support personnel having a 50% lower rate than the regular force (RR, 0.5; 95% CI, 0.5-0.6).

**Figure.**
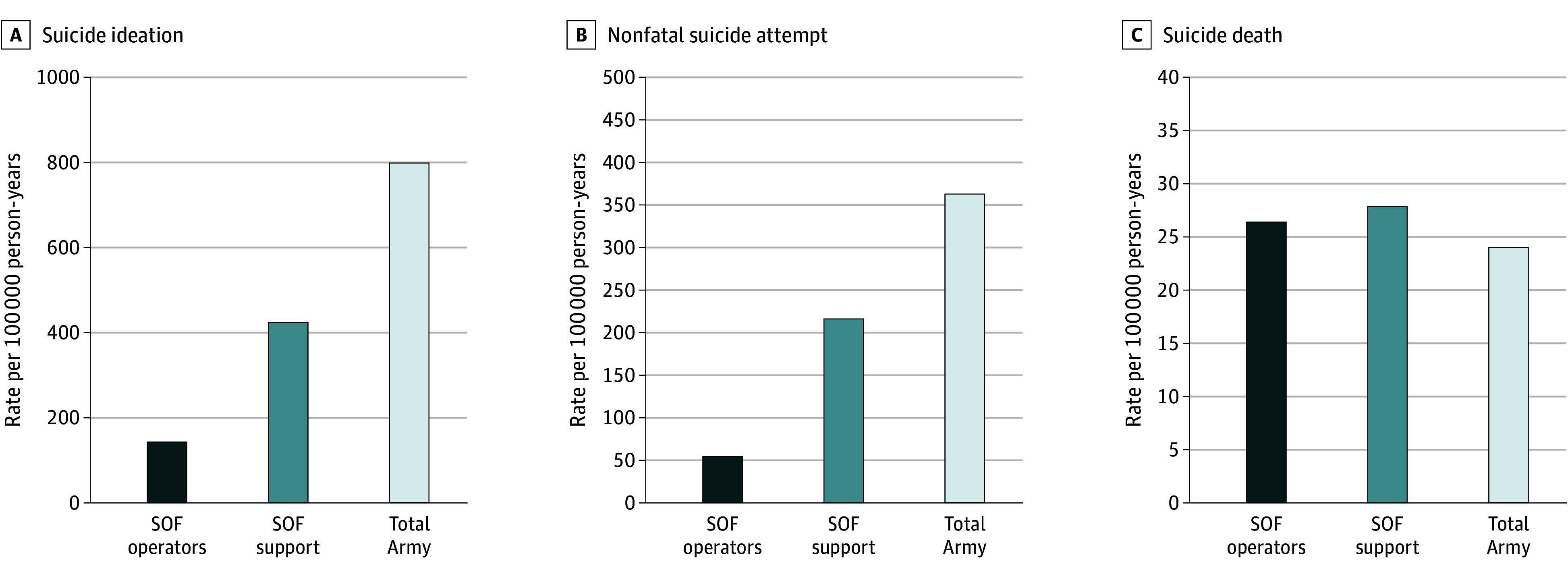
Rates of Suicide Ideation, Nonfatal Suicide Attempt, and Suicide Death Among Special Operations Forces (SOF) Operators, SOF Support, and the Total Regular Army Enlisted Force

Among operators, the rate of nonfatal suicide attempt was numerically lower during person-months when the soldiers were currently in a SOF unit vs not currently in a SOF unit (mean [SD], 45.7 [27.1] vs 87.5 [73.4] per 100 000 person-years). This was also true for suicide attempt among soldiers who were ever SOF support personnel (mean [SD], 149.7 [43.0] vs 302.5 [54.7] per 100 000 person-years). A similar pattern was observed for suicide death among current vs not current operators (mean [SD], 11.0 [15.8] vs 88.5 [79.6] per 100 000 person-years) and support personnel (mean [SD], 23.8 [14.7] vs 33.1 [20.1] per 100 000 person-years), and for suicide ideation (mean [SD], operators: 112.6 [65.2] vs 255.6 [117.9] per 100 000 person-years; mean [SD], support personnel: 282.8 [48.7] vs 588.0 [144.8] per 100 000 person-years).

### Ratio of Nonfatal Suicide Attempt to Suicide Death

The ratio of nonfatal suicide attempts to suicide deaths was 2.1 (95% CI, 1.9-2.3) for operators and 7.6 (95% CI, 7.5-7.7) for support personnel. The ratio was 15.0 (95% CI, 15.0-15.0) for the total regular force.

### Standardized Risk of Suicidal Behavior

When SREs were calculated based on a logistic regression model that included sociodemographic and Army career characteristics, the adjusted suicide death risk among operators still did not differ significantly from support personnel (RR, 1.1; 95% CI, 0.6-1.8) or the regular enlisted force (excluding SOF operators and support personnel) (RR, 1.1; 95% CI, 0.6-1.8), nor did risk differ between support personnel and the regular force (RR, 1.1; 95% CI, 0.8-1.5). When SREs were calculated for nonfatal suicide attempt, operators continued to have lower risk than support personnel (RR, 0.5; 95% CI, 0.4-0.7) and the regular force (excluding operators and support personnel) (RR, 0.3; 95% CI, 0.3-0.5), and risk among support personnel remained lower than the regular force (RR, 0.7; 95% CI, 0.6-0.8), although the magnitude of these differences were attenuated relative to the crude (unadjusted) rates. The same was true for risk of suicide ideation, which remained lower among operators than support personnel (RR, 0.6; 95% CI, 0.5-0.8) and the regular force (excluding operators and support personnel) (RR, 0.3; 95% CI, 0.3-0.4), and support personnel remained lower than the regular force (RR, 0.6; 95% CI, 0.5-0.6).

### Risk Factors for Suicide Attempt Among Operators and Support Personnel

In multivariable logistic regression analyses among operators, odds of suicide attempt were significantly higher among women (OR, 7.4; 95% CI, 3.2-17.1), those who were demoted in the past year (OR, 9.0; 95% CI, 3.5-23.1), and those who had received a mental health diagnosis (OR, 11.5; 95% CI, 5.9-22.3). Odds were lower among those who were higher ranking (E5-E6: OR, 0.3; 95% CI, 0.1-0.7; E7-E8: OR, 0.2; 95% CI, 0.1-0.6) (Table 2 and eTable 7 in Supplement 1).

**Table 2.** Multivariable Associations of Sociodemographic, Army Career, and Mental Health Characteristics With Nonfatal Suicide Attempt Among Regular Army Enlisted Special Operations Forces (SOF)^**a**^

Characteristics	SOF element						SOF element × characteristic interaction, χ^2^^b^	*P* value
	Operators			Support				
	OR (95% CI)	χ^2^	*P* value	OR (95% CI)	χ^2^	*P* value		
Sex								
Male	1 [Reference]	21.8^c^	<.001	1 [Reference]	1.4	.23	6.2^c^	.013^d^
Female	7.4 (3.2-17.1)			1.3 (0.9-2.0)				
Age, y								
≤24	1.3 (0.5-3.3)	4.1	.25	2.2 (1.4-3.3)	30.2^c^	<.001	0.9	.84
25-29	0.8 (0.4-1.7)			1.1 (0.7-1.6)				
30-34	1 [Reference]			1 [Reference]				
≥35	0.5 (0.2-1.2)			1.0 (0.6-1.8)				
Race or ethnicity								
White non-Hispanic	1 [Reference]	1.1	.29	1 [Reference]	8.5^c^	.004	0.2	.68
Other^e^	0.7 (0.3-1.4)			0.7 (0.5-0.9)				
Marital status								
Not currently married	0.8 (0.4-1.4)	0.8	.40	0.7 (0.6-0.9)	9.3^c^	.002	0.1	.78
Currently married	1 [Reference]			1 [Reference]				
Rank								
E1-E4	1 [Reference]	8.9^c^	.012	1 [Reference]	22.8^c^	<.001	1.3	.53
E5-E6	0.3 (0.1-0.7)			0.6 (0.4-0.8)				
E7-E8	0.2 (0.1-0.6)			0.2 (0.1-0.4)				
Deployment status								
Currently/previously deployed	1 [Reference]	0.2	.65	1 [Reference]	28.4^c^	<.001	1.3	.25
Never deployed	1.2 (0.6-2.5)			1.9 (1.5-2.4)				
Demoted in the past year								
Yes	9.0 (3.5-23.1)	21.1^c^	<.001	2.5 (1.8-3.5)	27.5^c^	<.001	5.1^c^	.02^d^
No^f^	1 [Reference]			1 [Reference]				
SOF status								
Currently SOF	1 [Reference]	1.5	.23	1 [Reference]	18.4^c^	<.001	0	.94
Not currently SOF	1.4 (0.8-2.5)			1.6 (1.3-2.1)				
Mental health diagnosis^g^								
Yes	11.5 (5.9-22.3)	52.0^c^	<.001	6.5 (5.1-8.3)	228.7^c^	<.001	3.1	.08
No	1 [Reference]			1 [Reference]				

Abbreviation: SOF, Special Operations Forces.

^a^ This sample of regular Army enlisted Special Operations Forces, which includes all person-months with a first suicide attempt (n = 48 operator cases; n = 333 support cases) and an equal-probability sample of controls person-months (unweighted n = 3173 operator controls; unweighted n = 14 166 support controls), is a subset of all soldiers in the 2004 through 2012 Army STARRS Historical Administrative Data Study (HADS). Control person-months were assigned a weight of 322.4 to adjust for undersampling.

^b^ Each 2-way interaction was examined in the total sample of SOF (including both operators and support soldiers) using a multivariable logistic regression model that adjusted for the main effects SOF role (operator vs support) and all other sociodemographic and Army career variables in the table.

^c^ *P* < .05.

^d^ Although statistically significant, these interactions include cell sizes among operators that are too small for reliable interpretation.

^e^ The Other race or ethnicity category includes American Indian or Alaska Native, Asian, Black, Hispanic, and Native Hawaiian or other Pacific Islander. eTable 3 in Supplement 1 contains a complete list of the original database categories.

^f^ Includes those who have never been demoted as well as those who were demoted more than a year ago.

^g^ Administratively documented mental health diagnosis.

In multivariable analyses among SOF support personnel, odds of suicide attempt were higher among soldiers who were age 24 years or younger (OR, 2.2; 95% CI, 1.4-3.3), never deployed (OR, 1.9; 95% CI, 1.5-2.4), demoted in the past year (OR, 2.5; 95% CI, 1.8-3.5), not currently SOF support personnel (OR, 1.6; 95% CI, 1.3-2.1), and those who had received a mental health diagnosis (OR, 6.5; 95% CI, 5.1-8.3). Odds were lower among support personnel who identified as having a race or ethnicity other than White non-Hispanic (OR, 0.7; 95% CI, 0.5-0.9), were not currently married (OR, 0.7; 95% CI, 0.6-0.9), and were higher ranking (E5-E6, OR, 0.6; 95% CI, 0.4-0.8; E7-E8, OR, 0.2; 95% CI, 0.1-0.4) (Table 2 and eTable 7 in Supplement 1). Two-way interactions indicated that sex (χ^2^ = 6.2; *P* = .01) and past-year demotion (χ^2^ = 5.1; *P* = .02) were the only suicide attempt risk factors that differed for operators vs support personnel, but their cell sizes among operators were too small for reliable interpretation. Comparable analyses of suicide ideation are available in eTables 7 and 8 in Supplement 1.

## Discussion

To our knowledge, the current study is the most comprehensive examination ever of suicidal behavior among the elite soldiers of US Army SOF during the Iraq and Afghanistan wars, and possibly the most comprehensive study of this kind during any SOF era. Analyses yielded 4 noteworthy findings. First, operators had a much lower risk of suicide ideation and nonfatal suicide attempt than other soldiers, including SOF support personnel. In contrast, operators had comparable rates of suicide death compared to both SOF support personnel and the total regular force. Second, SREs that adjusted for the sociodemographic and Army career differences between operators and other soldiers attenuated, but did not eliminate, group differences in risk of suicide ideation and nonfatal suicide attempt. Third, there were marked group differences in the ratio of nonfatal suicide attempts to suicide deaths, ranging from approximately 15-to-1 for the total regular enlisted force to 2-to-1 for operators. Fourth, multivariable analyses identified several differences between SOF operators and support personnel in risk factors for suicide attempt, with rank, past-year demotion, and mental health diagnosis emerging as the only factors that were significant in both groups. It is also notable that crude rates of all 3 suicide-related outcomes among those who had ever served as SOF operators or support personnel were numerically higher during months when those soldiers were not serving in SOF units, highlighting the importance of contextual factors, such as unit cohesion,^21,22^ that may protect against suicide risk.

In this study, the lower nonfatal suicide attempt risk among operators compared with other soldiers was true for the complete group of operators and not only the subgroup of Green Berets, which was previously documented.^7^ Operators also had lower risk of suicide attempt than support personnel, the other major element within the SOF community. The same pattern was observed for suicide ideation. Importantly, rates of suicide death did not differ between operators, support personnel, and the total enlisted regular Army. In fact, the ratio of nonfatal suicide attempts to suicide deaths was smaller among operators (2:1) than support personnel (7:1) and the total regular force (15:1). One interpretation of this is that operators are less likely than other soldiers to engage in suicidal behavior overall, but when they do, it is far more likely to be fatal.^23^ Although the reasons for this are not yet known, firearms access^24^ and capability for lethal self-injury^25,26^ are relevant risk factors that may differentiate operators from most other soldiers. Recent findings suggest that the true rate of nonfatal suicide attempt among soldiers may be much higher than what is indicated by administrative medical records,^27^ but additional research is needed to determine whether rates of undetected suicide attempt differ for SOF vs other soldiers and, if so, the degree to which such a difference accounts for the discrepant attempt-to-death ratios. However, it is notable that the rate of mental health diagnosis among operators (31.0%) is the same as that of SOF support personnel (31.6%) and only slightly lower than that of the total regular enlisted force (36.3%), suggesting that operators are generally as likely as other soldiers to interact with the Army’s mental health care system. In fact, among soldiers who made a suicide attempt, the rate of previous mental health diagnosis among operators (80.0%) exceeds that of support personnel (69.6%) and the total regular enlisted force (66.7%). Future research might examine the extent to which diagnostic rates are associated with aspects of embedded behavioral health that are unique to the SOF community.^28^

The similar risk of suicide death between operators and other soldiers is in stark contrast to operators’ substantially lower risk of suicide attempt, a discrepancy that may provide further support to evidence that fatal and nonfatal suicidal behaviors are largely distinct types of events carried out by distinct but overlapping populations.^29,30,31,32,33^ It also raises the important question of whether the differences in suicide attempt risk between operators and other soldiers are primarily attributable to population difference in sociodemographic and military characteristics known to be associated with suicidal behavior (eg, sex, age, rank).^2,13,34^ Using SREs that adjusted for a number of relevant characteristics, the significant differences in risk between operators and other soldiers persisted, with operators being 50% less likely than SOF support personnel and 70% less likely than other regular enlisted soldiers to have a suicide attempt. This suggests that the lower suicide attempt risk among operators must be due to unmeasured characteristics or experiences, even compared with support personnel who also serve in SOF units. For example, although operators are more likely than support personnel to report direct combat exposure, they score higher than support personnel on measures of resilience and social support, and also report higher quantity and quality of sleep.^9^ It would be beneficial for future research to consider a broad range of individual differences (eg, emotion reactivity and regulation, distress tolerance, and personality dimensions) in attempting to account for the remaining discrepancy in suicide attempt risk between operators and other soldiers.

In multivariable analyses, odds of a suicide attempt among operators were approximately 7 times higher for women, 9 times higher for those demoted in the past year, and 12 times higher for those who received a mental health diagnosis, whereas odds were 70% to 80% lower for soldiers with an enlisted rank of E5 or above. Among SOF support personnel, sex was the only sociodemographic or Army career characteristic not significantly associated with suicide attempt, although its association was in the same direction as operators (ie, higher odds among women). The other risk factors among SOF support personnel were largely consistent with those previously identified in the total regular Army enlisted population.^34^ If replicated in a larger sample, these findings would highlight the importance of understanding and addressing the unique experiences and challenges faced by women operators, such as ill-fitting equipment, sex bias, sexual harassment, isolation, and loneliness.^35^ Because of a major change in Army policy that lifted the ban on women serving in ground combat units approximately a decade ago,^36^ the necessity of such work continues to grow.

### Limitations

This study has limitations. First, whereas potential suicide death are thoroughly investigated by the Army and there is little evidence of misclassification,^37^ administrative medical records are unlikely to capture all suicide attempt due to factors, such as underreporting and errors in clinician diagnosis and coding. Recent work indicates that many self-reported suicide attempt in the Army, as in civilian populations, are not administratively documented.^27^ This discrepancy is likely even more extreme for suicide ideation, which is rarely endorsed by soldiers during mental health screening,^38^ and the assessment of which is not standardized across clinicians. Because of this, our findings regarding suicide attempt and suicide ideation could have been affected by significant differences in health care use and/or delivery among operators, SOF Support personnel, and other soldiers, although we are not aware of data demonstrating such differences. The extent to which there are undocumented suicidal thoughts and behaviors in SOF is unknown. Given the findings of this analysis, it may be valuable to carry out such research to develop a more complete picture of suicidal events in the SOF population. Second, we were unable to examine risk factors for suicide death due to the small number of suicide death among operators (n = 21). Such analyses may be possible in the future by extending the length of the study period or by expanding to include operators from other US military services (US Marine Corps, Navy, Air Force, and Space Force). Third, our findings may not generalize to other military eras or conflicts, or to other current or former SOF personnel. Most significantly, although comprehensive of regular Army enlisted SOF personnel, this study excluded officers and National Guard and Reserve soldiers who are important members of SOF units and warrant targeted research.

## Conclusions

In this retrospective cohort study of US regular Army enlisted soldiers during the wars in Iraq and Afghanistan, SOF operators were less likely than other soldiers to engage in suicidal behavior, but when they did it was more likely to be fatal. The current study extends previous SOF research by considering a range of major suicide-related outcomes (suicide ideation, suicide attempt, and suicide death) and fully representing enlisted regular Army operators. To our knowledge, it is also the first study examining risk for suicidal thoughts and behaviors among SOF support personnel. The findings demonstrate the need for improved understanding of risk in the SOF community, particularly the higher proportion of fatal suicidal behaviors among operators.

## Notes

Supplement 1.
eMethods.
**eTable 1.** List and brief descriptions of administrative data systems in the Army STARRS Historical Administrative Data Study (HADS) included in the current study **eTable 2.** List of military occupational codes categorized as regular Army Special Operations Forces Operators **eTable 3.** Original race and ethnicity categories in US Army and Department of Defense Data systems included in the Army STARRS Historical Administrative Data Study (HADS) **eTable 4. ***International Classification of Diseases, Ninth Revision–Clinical Modification* (*ICD-9-CM*) Codes Used to Identify Mental Disorders **eTable 5. **Distribution of sample characteristics by suicide death among SOF Operators, SOF Support, and the total Regular Army enlisted force, 2004-2012 **eTable 6. **Distribution of sample characteristics by suicide ideation among SOF Operators, SOF Support, and the total Regular Army enlisted force, 2006-2012 **eTable 7.** Univariable associations of sociodemographic, Army career, and mental health characteristics with suicide attempts among regular Army enlisted Special Operations Forces (SOF) **eTable 8.** Univariable associations of sociodemographic, Army career, and mental health characteristics with suicide ideation among regular Army enlisted Special Operations Forces (SOF) **eTable 9.** Multivariable associations of sociodemographic, Army career, and mental health characteristics with suicide ideation among regular Army enlisted Special Operations Forces (SOF)
eReferences.

Supplement 2.
Data Sharing Statement
