# Supplementary material for: Suicidal Behavior in US Army Special Operations Forces
Source: JAMA Netw Open. 2025 Aug 15;8(8):e2527395. doi: 10.1001/jamanetworkopen.2025.27395 (PMC12357185; doi:10.1001/jamanetworkopen.2025.27395)
Supplement: Supplement 1. — eMethods. eTable 1. List and brief descriptions of administrative data systems in the Army STARRS Historical Administrative Data Study (HADS) included in the current study eTable 2. List of military occupational codes categorized as regular Army Special Operations Forces Operators eTable 3. Original race and ethnicity categories in US Army and Department of Defense Data systems included in the Army STARRS Historical Administrative Data Study (HADS) eTable 4. International Classification of Diseases, Ninth Revision–Clinical Modification (ICD-9-CM) Codes Used to Identify Mental Disorders eTable 5. Distribution of sample characteristics by suicide death among SOF Operators, SOF Support, and the total Regular Army enlisted force, 2004-2012 eTable 6. Distribution of sample characteristics by suicide ideation among SOF Operators, SOF Support, and the total Regular Army enlisted force, 2006-2012 eTable 7. Univariable associations of sociodemographic, Army career, and mental health characteristics with suicide attempts among regular Army enlisted Special Operations Forces (SOF) eTable 8. Univariable associations of sociodemographic, Army career, and mental health characteristics with suicide ideation among regular Army enlisted Special Operations Forces (SOF) eTable 9. Multivariable associations of sociodemographic, Army career, and mental health characteristics with suicide ideation among regular Army enlisted Special Operations Forces (SOF) eReferences. [file jamanetwopen-e2527395-s001.pdf]

Supplemental Online Content

Naifeh JA, Ursano RJ, Shor R, et al. Suicidal behavior in US Army Special Operations Forces. *JAMA Netw Open*. 2025;8(8):e2527395. doi:10.1001/jamanetworkopen.2025.27395

eMethods.
eTable 1. List and brief descriptions of administrative data systems in the Army STARRS Historical Administrative Data Study (HADS) included in the current study
eTable 2. List of military occupational codes categorized as regular Army Special Operations Forces Operators
eTable 3. Original race and ethnicity categories in US Army and Department of Defense Data systems included in the Army STARRS Historical Administrative Data Study (HADS)
eTable 4. *International Classification of Diseases, Ninth Revision–Clinical Modification* (*ICD-9-CM*) Codes Used to Identify Mental Disorders
eTable 5. Distribution of sample characteristics by suicide death among SOF Operators, SOF Support, and the total Regular Army enlisted force, 2004-2012
eTable 6. Distribution of sample characteristics by suicide ideation among SOF Operators, SOF Support, and the total Regular Army enlisted force, 2006-2012
eTable 7. Univariable associations of sociodemographic, Army career, and mental health characteristics with suicide attempts among regular Army enlisted Special Operations Forces (SOF)
eTable 8. Univariable associations of sociodemographic, Army career, and mental health characteristics with suicide ideation among regular Army enlisted Special Operations Forces (SOF)
eTable 9. Multivariable associations of sociodemographic, Army career, and mental health characteristics with suicide ideation among regular Army enlisted Special Operations Forces (SOF)
eReferences.

This supplemental material has been provided by the authors to give readers additional information about their work.

© 2025 Naifeh JA et al. *JAMA Network Open*.

SUPPLEMENTAL MATERIALS

eMethods

Sample

The Army STARRS Historical Administrative Data Study (HADS) is a longitudinal, retrospective cohort study that integrates 40 Army and Department of Defense (DoD) administrative data systems, including every system that documents suicidal events (eTable 1).[12] Within the HADS are individual-level person-month records for all Regular Army enlisted soldiers who were on active duty at some point from January 1, 2004 through December 31, 2012 (*N*=1,070,885 soldiers). For soldiers who were on active duty during this period but began service prior to 2004, records dated back to January 1, 2000. Person-month records were created by coding each month of a soldier's career separately for each administrative variable and allowing values to change over time.[13,14] The current study focused on Regular Army enlisted soldiers, excluding officers, Army National Guard, and Army Reserve, which differ from Regular Army enlisted soldiers in military career experiences, sociodemographic characteristics, and risk for suicidal behavior.[13-15] In addition, National Guard and Reserve soldiers typically do not have access to military healthcare during periods when they are deactivated from federal service, substantially limiting the capture of SI and SA in those populations. We selected three independent case-control samples, one for each suicide-related outcome (nonfatal suicide attempt [SA], suicide death [SD], and suicide ideation [SI]). The analytic sample for nonfatal SA included all 14,547 person-months in which a first SA occurred during 2004-2012 and an equal-probability sample of 149,166 control person-months in which a documented SA did not occur (person-months in which a soldier died were excluded). Data were analyzed using a discrete-time survival framework with person-month as the unit of analysis. [13,14] Discrete-time survival coefficients can be estimated without bias when control person-months are randomly subsampled and weighted using the logic of case-control analysis. [33] Therefore, we used a 1:20 case:control ratio to reduce computational intensity, selecting an equal-probability sample of control person-months after stratifying the population by sex, rank, time in service, and deployment status. Each control person-month was assigned a weight of 322.4 (the inverse probability of selection) to adjust for under-sampling.

© 2025 Naifeh JA et al. *JAMA Network Open*.

An identical process was used to create the analytic samples for SD (*n*=972 cases, *n*=15,630 control person-months, each assigned a weight of 3,111.2) and SI (*n*=25,584 cases; *n*=278,708 control person-months, each assigned a weight of 137.9), the latter of which was not administratively documented until 2006 (i.e., a 2006-2012 study period). The Army STARRS HADS was approved by the Institutional Review Board of the University of Michigan Institute for Social Research, with secondary review and approval by the Uniformed Services University, University of California–San Diego, and Harvard Medical School, all of which determined that the present study did not constitute human participant research because it relies entirely on deidentified secondary data.

Identifying Special Operations Forces (SOF). We worked with U.S. Army Special Operations subject matter experts to identify special operations units based on their unit identification codes (UICs), which are alphanumeric codes used to uniquely identify and track units, assign personnel, and manage logistics. Person-months were identified in which either the soldier's assigned unit or duty unit had a special operations UIC. Once person-months associated with special operations UICs were identified, Regular Army enlisted soldiers within those units were separated into Operators (eTable 2) and Support personnel based on military occupational specialty (MOS) codes from the Defense Manpower Data Center (DMDC). Each person month was coded to indicate whether the soldier was currently or not currently an Operator/Support soldier. The primary analyses (described below) examined suicide-related outcomes that occurred at any point during service after a soldier first became an Operator or SOF Support.

Measures

Suicidal Thoughts and Behaviors. SDs, determined following thorough investigation by the Army's Criminal Investigation Division, were identified using the Armed Forces Medical Examiner Tracking System, which contains data on all suicides that occur while on active duty. Nonfatal SAs, which are diagnosed based on clinician judgment, were identified using administrative records from: the DoD Suicide Event Report (DoDSER),[15] a DoD-wide surveillance mechanism that aggregates information on suicidal behavior via a standardized form completed by medical providers; and ICD-9-CM codes E950-E958 (indicating self-inflicted poisoning or injury with suicidal intent)[34] from the Military Health System Data Repository (MDR), Theater

© 2025 Naifeh JA et al. *JAMA Network Open*.

Medical Data Store (TMDS), and TRANSCOM (Transportation Command) Regulating and Command and Control Evacuating System (TRAC^{2}ES), which together provide healthcare encounter information from military and civilian treatment facilities, combat operations, and aeromedical evacuations. Administrative documentation of SI, also diagnosed based on clinician judgement, was identified using DoDSER records and the ICD-9-CM code V62.84 from MDR, TMDS, and TRAC^{2}ES (eTable 1).

Sociodemographic and Army career characteristics. Administrative personnel records were used to derive sociodemographic variables (sex, age, marital status, and race-ethnicity dichotomized as White non-Hispanic vs. other [American Indian/Alaska Native, Asian, Black, Hispanic, and Native Hawaiian or other Pacific Islander] owing to small cell sizes for some outcomes; see eTable 3 for original database categories]) and Army career variables (military occupational specialty [SOF Operator, SOF Support, and all other enlisted soldiers], rank, deployment status, and past-year demotion).

Mental Health Diagnosis. Administrative medical data were used to create an indicator variable for previous mental health diagnosis during Army service by combining categories derived from ICD-9-CM mental disorder codes (e.g., major depression, bipolar disorder, posttraumatic stress disorder, personality disorders), including V-codes for stressors/adversities and marital problems (eTable 4).

Missing Values

During creation of the HADS integrated database, some item-level administrative data were missing for particular person-months. In most cases, these data could be recovered by cross-checking other data systems or other months in the same soldiers' records. The few missing values that could not be recovered through cross-checking were assigned imputed values equal to subgroup modes.

Analysis Methods

Analyses were conducted using SAS version 9.4. [16] The weighted samples were used in all analyses. Crude rates of SI, nonfatal SA, and SD were calculated for Operators, Support, and the total Regular Army enlisted population, then compared with risk ratios (RRs). We then used the coefficients from multivariable logistic regression models that adjusted for group differences in sociodemographic and Army career variables (sex, age, race-ethnicity, marital status, rank, deployment status) to generate standardized risk estimates (SREs)

© 2025 Naifeh JA et al. *JAMA Network Open*.

[17] for SI, SA, and SD (per 100,000 person-years) among Operators, Support, and all other Regular enlisted soldiers. Each SRE is an estimate of the risk of the outcome expected under the model if the distributions of all other predictors were the same for Operators, Support, and other Regular enlisted soldiers. Logistic regression analyses, conducted separately among Operators and Support, examined associations of sociodemographic, Army career, and mental health diagnosis variables with SA in a given person-month, with all risk factors other than sex and race-ethnicity treated as time-varying covariates. The combined sample of Operators and Support was used to examine two-way interactions between SOF element (Operator vs. Support) and the other predictor variables. Each interaction was examined in a separate multivariable model that adjusted for the main effects of all other variables. The same approach was used to examine risk factors for SI. Logistic regression coefficients were exponentiated to obtain odds-ratios (OR) and 95% confidence intervals (CI). All logistic regression models included a dummy predictor for calendar month and year to control for secular trends. Coefficients of other predictors can consequently be interpreted as averaged within-month associations.

© 2025 Naifeh JA et al. *JAMA Network Open*.

eTable 1. List and Brief Descriptions of Administrative Data Systems in the Army STARRS Historical Administrative Data Study (HADS) Included in the Current Study

Database Acronym	Description
AFMETS	ARMED FORCES MEDICAL EXAMINER TRACKING SYSTEM (AFMETS): Variables include manner of death and cause of death, including self-inflicted.
DMDC/CTS	DEFENSE MANPOWER DATA CENTER (DMDC) / CONTINGENCY TRACKING SYSTEM (CTS): Collection of activation, mobilization, and deployment data. Provides information to DoD decision makers and includes a CTS Deployment File used for tracking the location of deployed personnel.
DMDC/Master Personnel & DMDC/Transaction files	DEFENSE MANPOWER DATA CENTER (DMDC) / MASTER PERSONNEL & TRANSACTION FILES: The Active Duty Master File provides an inventory of all individuals on active duty (excluding reservists on active duty for training) at a point in time. It is a standardized and centralized database of present and past members of the active duty force. Personal data elements include social security number, education level, home of record, date of birth, marital status, number of dependents, race, ethnic group, and name. Military data elements include Service, pay grade, Armed Forces Qualification Test percentile (enlisted only), source of commission (officers only), military primary duty and secondary occupation, Unit Identification Code, months of service, duty location, Estimated Termination of Service date, basic active service date, date of current rank, pay entry base date, foreign language ability, and major command code.
DODSER	DEPARTMENT OF DEFENSE SUICIDE EVENT REPORT (DODSER): Provides risk and protective factor information for suicide events. This file contains suicidal ideation, non-fatal attempts, and completed suicides.
MDR	MILITARY HEALTH SYSTEM DATA REPOSITORY (MDR): This database contains information about medical, dental, pharmaceutical, and ancillary claims data for both in network and purchased care as well as both inpatient and outpatient treatment. Data are collected on both military personnel and their beneficiaries.
TMDS	THEATER MEDICAL DATA STORE (TMDS): Used to track, analyze, view and manage Soldier medical treatment information recorded in the theater of operations. Features of TMDS: accessibility and visibility of service members' deployed medical records, outpatient and inpatient treatment records created in theater facilities, treatment records from other applications, reports on movement of patients, patient status and injuries/illnesses.
TRAC2ES	TRANSCOM REGULATING AND COMMAND & CONTROL EVACUATION SYSTEM (TRAC2ES): A tracking system for all medical transfers across the world for all DOD services.

© 2025 Naifeh JA et al. *JAMA Network Open*.

eTable 2. List of Military Occupational Codes Categorized as Regular Army Special Operations Forces Operators

Operator MOS/AOC Codes	Description
18A*, 180A*, 18B, 18C, 18D, 18E, 18F, 18Z	Special Forces Green Beret
37A*, 37B, 37F	Military Information Support Operations
38A*, 38B	Civil Affairs
11A*, 11B and 11C, 11Z with “G” or "V" SQI	Rangers, "5S", "5R" SI for officers (11A)
15A*, 15B, 152C*, 153E*, 154E*, with "K4" or “K5” OR “K6” ASI or SI	Special Operators Aviation Regiment (SOAR)

*Indicates Officer or Warrant Officer, which were not included in the current study.
MOS = Military Occupational Specialty
AOC = Area of Concentration
SQI = Special Qualifications Identifier
ASI = Additional Skill Identifier
SI = Skill Identifier

© 2025 Naifeh JA et al. *JAMA Network Open*.

eTable 3. Original Race and Ethnicity Categories in U.S. Army and Department of Defense Data Systems Included in the Army STARRS Historical Administrative Data Study (HADS)

**Race Categories**
American Indian/Alaska Native
Asian
Black
Native Hawaiian or other Pacific Islander
White
Unknown
**Ethnicity Categories**
Aleut
Chinese
Cuban
Eskimo
Filipino
Guamanian
Indian
Japanese
Korean
Latin American
Melanesian
Mexican
Micronesian
North American Indian
Other Asian
Other Hispanic
Other Pacific Islander
Polynesian
Puerto Rican
Vietnamese
Unknown

© 2025 Naifeh JA et al. *JAMA Network Open*.

eTable 4: International Classification of Diseases, Ninth Revision–Clinical Modification (ICD-9-CM) Codes Used to Identify Mental Disorders

Included Mental Health Diagnoses	ICD-9-CM Codes
Adjustment Disorder	309, .29, .3, .4, .82, .83, .89, .9
Dysthymic Disorder/Neurasthenia/Depression NOS	296.82, .90, .99<br>300.4, .5<br>309.0, .1<br>311, .0, .1<br>313.1
Major Depression	296.2, .20, .21, .22, .23, .24, .25, .26, .3, .30, .31, .32, .33, .34, .35, .36
Bipolar Disorder	296.00, .01, .02, .03, .04, .05, .06, .10, .11, .12, .13, .14, .15, .16, .40, .41, .42, .43, .44, .45, .46, .50, .51, .52, .53, .54, .55, .56, .60, .61, .62, .63, .64, .65, .66, .7, .80, .81, .89<br>301.13
Anxiety State/Anxiety Disorder	300, .00, .01, .02, .09, .20, .21, .22, .23, .29, .3<br>309.21, .24, .28<br>313.0, .21, .22, .23
Post-Traumatic Stress Disorder	309.81
ADHD/Learning Disorders	314.0, .00, .01, .1, .2, .8, .9<br>315.00, .01, .02, .09, .1, .2, .3, .31, .32, .34, .39, .4, .5, .8, .9
Conduct Disorder/ Oppositional Defiant Disorder	301.7<br>312.4, .8, .81, .82, .89, .9<br>313.81<br>V62.83
Eating Disorders	307.1, .50, .51, .59

© 2025 Naifeh JA et al. *JAMA Network Open*.

© 2025 Naifeh JA et al. *JAMA Network Open*.

Other Impulse Control Disorders	312.00, .01, .02, .03, .10, .11, .12, .13, .20, .21, .22, .23, .3, .30, .31, .32, .33, .34, .35, .39
Alcohol Induced Mental Disorders/Alcohol Dependence/Alcohol Abuse	291.0, .1, .2, .3, .4, .5, .8, .81, .82, .89, .9<br><br>303.00, .01, .02, .03, .9, .90, .91, .92, .93<br><br>305, .0, .00, .01, .02, .03
Drug Induced Mental Disorders	292
Non-Dependent Drug Abuse	305.2, .20, .21, .22, .23, .3, .30, .31, .32, .33, .4, .40, .41, .42, .43, .5, .50, .51, .52, .53, .6, .60, .61, .62, .63, .7, .70, .71, .72, .73, .8, .80, .81, .82, .83, .9, .90, .91, .92, .93
Drug dependence	304
Personality Disorders	301.0, .1, .10, .11, .12, .20, .21, .22, .3, .4, .50, .51, .59, .6, .8, .80, .81, .82, .83, .84, .89, .9
Non-Affective Psychosis	295.00, .01, .02, .03, .04, .05, .10, .11, .12, .13, .14, .15, .20, .21, .22, .23, .24, .25, .30, .31, .32, .33, .34, .35, .40, .41, .42, .43, .44, .45, .50, .51, .52, .53, .54, .60, .61, .62, .63, .64, .65, .70, .71, .72, .73, .74, .75, .80, .81, .82, .83, .84, .85, .90, .91, .92, .93, .94, .95<br><br>297.0, .1, .2, .3, .8, .9<br><br>298.0, .1, .2, .3, .4, .8, .9, .90
Somatoform/Dissociative Disorders	300.10, .11, .12, .13, .14, .15, .16, .19, .6, .7, .80, .81, .82, .89<br><br>306.0, .1, .2, .3, .4, .50, .51, .52, .53, .59, .6, .7, .8, .9<br><br>307.54, .80, .81, .89
Organic Mental Disorders	290.0, .10, .11, .12, .13, .20, .21, .3, .40, .41, .42, .43, .8, .9<br><br>293.0, .1, .81, .82, .83, .84, .89, .89, .9<br><br>294.0, .1, .10, .11, .8, .9<br><br>307.20, .21, .22, .23, .3<br><br>310.0, .8, .9<br><br>317<br><br>318.0, .1, 2<br><br>319

© 2025 Naifeh JA et al. *JAMA Network Open*.

Sexual Disorders	302, .0, .1, .2, .3, .4, .50, .51, .52, .53, .6, .70, .71, .72, .73, .74, .75, .76, .79, .81, .82, .83, .84, .85, .89, .9
Sleep Disorders	307.4, .40, .41, .42, .43, .44, .45, .46, .47, .48, .49
Other Mental Disorders/Mental Illness	292.85
	299.00, .01, .10, .80, .81, .90, .91
	300.9
	307.0, .52, .53, .6, .7, .9
	309.22
	310.1
	313.3, .82, .89, .9
	316
Traumatic Stress	308, .0, .1, .2, .3, .4, .9
Stressors/Adversities	V40.0, .00, .1, .2, .20, .3, .30, .9, .90
	V61, .0, .01, .02, .03, .04, .05, .06, .07, .08, .09, .2, .20, .21, .22, .23, .24, .29, .3, .4, .41, .42, .49, .8, .9
	V62, .0, .1, .1 0, .2, .20, .21, .22, .29, .3, .4, .5, .8, 80, .81, .810, .811, .812, .82, .89, .9, .90
	V69.4, .5, .9
Marital Problems	V61.1, .10, .11, .12

**Excluded Mental Health Diagnoses**	**ICD-9-CM Codes**
Postconcussion Syndrome	310.2
Tobacco Use Disorder	305.1, .10, .11, .12, .13
Symptoms, Signs, and Ill-Defined Conditions, Mental	797
	798, .0, .1, .2, .9
	799, .0, .01, .02, .1, .2, .21, .22, .23, .24, .25, .29, .3, .4, .8, .81, .82, .89, .9

© 2025 Naifeh JA et al. *JAMA Network Open*.

Prior History of Mental Disorders	V11.0, .1, .2, .3, .8, .80, .9, .90
	V66.3
	V67.3
Indicator of Impulsivity and Risky Behavior	V69.2, .3
Self-Damaging Behavior	V69.8

© 2025 Naifeh JA et al. *JAMA Network Open*.

eTable 5. Distribution of sample characteristics by suicide death among SOF Operators, SOF Support, and the total Regular Army enlisted force, 2004–2012.[a]

	SOF Operators				SOF Support				All Regular Enlisted Soldiers			
	Suicide death cases		Total Population[b]		Suicide death cases		Total Population[b]		Suicide death cases		Total Population[b]	
	N[c]	%[c]	N	%	N[c]	%[c]	N	%	N	%	N	%
Sex												
Male	–	–	939,607	98.4	–	–	1,832,549	96.6	924	95.1	41,717,105	86.7
Female	–	–	15,556	1.6	–	–	62,224	3.4	48	4.9	6,386,084	13.3
Age												
≤ 24 years	–	–	158,675	16.6	–	–	777,824	41.0	472	48.6	20,450,621	42.5
25 – 29 years	–	–	286,238	30.0	–	–	507,142	27.0	271	27.9	11,812,581	24.6
30 – 34 years	–	–	239,569	25.1	–	–	311,125	16.4	99	10.2	6,975,214	14.5
≥ 35 years	–	–	270,682	28.3	–	–	298,681	15.6	130	13.4	8,864,773	18.4
Race-ethnicity												
White non-Hispanic	–	–	743,597	77.9	29	65.9	1,232,070	65.0	684	70.4	28,713,105	59.7
Other[d]	–	–	211,567	22.1	15	34.1	662,703	35.0	288	29.6	19,390,085	40.3
Marital status												
Not currently married	–	–	320,459	33.6	18	40.9	920,937	48.6	460	47.3	22,770,052	47.3
Currently married	–	–	634,705	66.4	26	59.1	973,836	51.4	512	52.7	25,333,137	52.7
Rank												
E1 – E4	–	–	87,117	9.1	21	47.7	858,716	45.3	633	65.1	26,690,671	55.5
E5 – E6	–	–	423,133	44.3	18	40.9	749,820	39.6	254	26.1	15,512,015	32.2
E7 – E8	–	–	444,914	46.6	5	11.4	286,237	15.1	85	8.8	5,900,503	12.3
Deployment status												
Currently/previously deployed	–	–	827,603	86.6	35	79.5	1,344,079	70.9	684	70.4	29,655,335	61.6
Never deployed	–	–	127,561	13.4	9	20.5	550,694	29.1	288	29.6	18,247,853	37.9
Demoted in the past year												
Yes	–	–	9,335	1.0	–	–	46,670	2.5	70	7.2	1,525,940	3.2
No[e]	–	–	945,829	99.0	–	–	1,848,103	97.5	902	92.8	46,577,246	96.8
Mental health diagnosis[f]												
Yes	–	–	336,025	35.2	22	50.0	622,265	32.8	615	63.3	17,437,748	36.3
No	–	–	619,138	64.8	22	50.0	1,272,508	67.2	357	36.7	30,665,441	63.7
**Total**	21	100	955,164	100	44	100	1,894,773	100	972	100	48,103,189	100

[a] This sample of Regular Army enlisted soldiers, which includes all person-months with a suicide death (n = 972 cases) and an equal-probability sample of controls person-months (unweighted n =15,630 controls), is a subset of all soldiers in the 2004–2012 Army STARRS Historical Administrative Data Study (HADS). Control person-months were assigned a weight of 3,111.2 to adjust for under-sampling.

© 2025 Naifeh JA et al. *JAMA Network Open*.

[b] Total Population includes both cases (i.e., person-months with a suicide death) and weighted control person-months.
[c] Data was omitted whenever a variable had cell sizes small enough to risk identification (n < 5).
[d] The "other" race-ethnicity category includes: American Indian/Alaska Native, Asian, Black, Hispanic, and Native Hawaiian or other Pacific Islander. For a complete list of the original database categories, see eTable 3.
[e] Includes those who have never been demoted as well as those who were demoted more than a year ago.
[f] Administratively documented mental health diagnosis.
SOF = Special Operations Forces

© 2025 Naifeh JA et al. *JAMA Network Open*.

eTable 6. Distribution of sample characteristics by suicide ideation among SOF Operators, SOF Support, and the total Regular Army enlisted force, 2006–2012.[a]

	SOF Operators				SOF Support				All Regular Enlisted Soldiers			
	Suicide ideation cases		Total Population[b]		Suicide ideation cases		Total Population[b]		Suicide ideation cases		Total Population[b]	
	N	%	N	%	N	%	N	%	N	%	N	%
Sex												
Male	92	94.8	806,127	98.4	539	94.9	1,538,995	95.7	21,052	82.3	33,439,457	87.0
Female	5	5.2	13,101	1.6	29	5.1	69,646	4.3	4,532	17.7	5,007,271	13.0
Age												
≤ 24 years	20	20.6	123,675	15.1	295	51.9	646,281	40.2	15,100	59.0	16,047,989	41.7
25 – 29 years	23	23.7	252,159	30.8	157	27.6	466,243	29.0	5,682	22.2	9,656,874	25.1
30 – 34 years	28	28.9	194,679	23.8	59	10.4	241,442	15.0	2,539	9.9	5,576,960	14.5
≥ 35 years	26	26.8	248,715	30.4	57	10.0	254,674	15.8	2,263	8.9	7,164,906	18.6
Race-ethnicity												
White non-Hispanic	76	78.4	652,128	79.6	425	74.8	1,088,648	67.7	17,604	68.8	23,189,838	60.3
Other[c]	21	21.6	167,101	20.4	143	25.2	519,992	32.3	7,980	31.2	15,256,890	39.7
Marital status												
Not currently married	42	43.3	279,197	34.1	267	47.0	725,106	45.1	13,115	51.3	17,685,369	46.0
Currently married	55	56.7	540,031	65.9	301	53.0	883,535	54.9	12,469	48.7	20,761,359	54.0
Rank												
E1 – E4	20	20.6	87,695	10.7	378	66.5	740,932	46.1	20,353	79.5	21,428,187	55.7
E5 – E6	42	43.3	334,477	40.8	173	30.5	650,019	40.4	4,550	17.8	12,347,626	32.1
E7 – E8	35	36.1	397,056	48.5	17	3.0	217,689	13.5	681	2.7	4,670,915	12.1
Deployment status												
Currently/previously deployed	85	87.6	722,718	88.2	395	69.5	1,225,783	76.2	13,143	51.4	24,986,032	65.0
Never deployed	12	12.4	96,510	11.8	173	30.5	382,857	23.8	12,441	48.6	13,460,696	35.0
Demoted in the past year												
Yes	6	6.2	4,969	0.6	67	11.8	40,734	2.5	2,819	11.0	1,242,131	3.2
No[d]	91	93.8	814,260	99.4	501	88.2	1,567,906	97.5	22,765	89.0	37,204,597	96.8
Mental health diagnosis[e]												
Yes	74	76.3	263,514	32.2	427	75.2	553,361	34.4	17,828	69.7	15,136,052	39.4
No	23	23.7	555,714	67.8	141	24.8	1,055,279	65.6	7,756	30.3	23,310,676	60.6
**Total**	97	100	819,228	100	568	100	1,608,640	100	25,584	100	38,446,728	100

[a] This sample of Regular Army enlisted soldiers, which includes all person-months with a first suicide ideation (n = 25,584 cases) and an equal-probability sample of controls person-months (unweighted n = 278,708 controls), is a subset of all soldiers in the 2006–2012 Army STARRS Historical Administrative Data Study (HADS). Control person-months were assigned a weight of 137.9 to adjust for under-sampling.

© 2025 Naifeh JA et al. *JAMA Network Open*.

[b] Total Population includes both cases (i.e., person-months with a first documented suicide ideation) and weighted control person-months.
[c] The "other" race-ethnicity category includes: American Indian/Alaska Native, Asian, Black, Hispanic, and Native Hawaiian or other Pacific Islander. For a complete list of the original database categories, see eTable 3.
[d] Includes those who have never been demoted as well as those who were demoted more than a year ago.
[e] Administratively documented mental health diagnosis
SOF = Special Operations Forces

© 2025 Naifeh JA et al. *JAMA Network Open*.

eTable 7. Univariable Associations of sociodemographic, Army career, and mental health characteristics with suicide attempts among Regular Army enlisted Special Operations Forces (SOF).[a]

	SOF Element			
	Operators		Support	
Characteristics	OR	(95% CI)	OR	(95% CI)
Sex				
Male	1.0	–	1.0	–
Female	15.0	(7.2–31.5)	1.6	(1.1–2.4)
χ^2_1 =	51.8* (p < .0001)		4.9* (p = .027)	
Age				
≤ 24 years	2.3	(1.1–4.8)	2.6	(1.8–3.8)
25 – 29 years	1.2	(0.6–2.6)	1.2	(0.8–1.9)
30 – 34 years	1.0	–	1.0	–
≥ 35 years	0.5	(0.2–1.2)	0.7	(0.4–1.2)
χ^2_3 =	14.3* (p = .003)		70.6* (p < .0001)	
Race-ethnicity				
White non-Hispanic	1.0	–	1.0	–
Other[b]	0.8	(0.4–1.7)	0.6	(0.5–0.8)
χ^2_1 =	0.2 (p = .63)		12.8* (p = .0003)	
Marital status				
Not currently married	1.3	(0.8–2.2)	1.0	(0.8–1.3)
Currently married	1.0	–	1.0	–
χ^2_1 =	0.8 (p = .37)		0.1 (p = .70)	
Rank				
E1 – E4	1.0	–	1.0	–
E5 – E6	0.3	(0.2–0.5)	0.4	(0.3–0.6)
E7 – E8	0.2	(0.1–0.3)	0.1	(0.1–0.3)
χ^2_2 =	28.7* (p < .0001)		75.5* (p < .0001)	
Deployment status				
Currently/previously deployed	1.0	–	1.0	–
Never deployed	1.7	(0.9–3.3)	2.0	(1.6–2.5)
χ^2_1 =	2.4 (p = .12)		40.7* (p < .0001)	
Demoted in the past year				
Yes	17.5	(7.5–40.7)	6.4	(4.6–8.8)
No[c]	1.0	–	1.0	–
χ^2_1 =	43.9* (p < .0001)		127.8* (p < .0001)	
SOF status				
Currently SOF	1.0	–	1.0	–
Not currently SOF	2.0	(1.1–3.4)	2.0	(1.6–2.5)
χ^2_1 =	5.4* (p = 0.020)		37.8* (p < .0001)	
Mental health diagnosis[d]				
Yes	8.9	(4.6–17.1)	5.2	(4.1–6.5)
No	1.0	–	1.0	–
χ^2_1 =	42.7* (p < .0001)		192.7* (p < .0001)	

[a] This sample of Regular Army enlisted Special Operations Forces, which includes all person-months with a first suicide attempt (n = 48 Operator cases; n = 333 Support cases) and an equal-probability sample of controls person-months (unweighted n = 3,173 Operator controls; unweighted n = 14,166 Support controls), is a subset of all soldiers in the 2004–2012 Army STARRS Historical Administrative Data Study (HADS). Control person-months were assigned a weight of 322.4 to adjust for under-sampling.

[b] The "other" race-ethnicity category includes: American Indian/Alaska Native, Asian, Black, Hispanic, and Native Hawaiian or other Pacific Islander. For a complete list of the original database categories, see eTable 3.

[c] Includes those who have never been demoted as well as those who were demoted more than a year ago.

[d] Administratively documented mental health diagnosis

SOF = Special Operations Forces

*p < .05

© 2025 Naifeh JA et al. *JAMA Network Open*.

eTable 8. Univariable Associations of sociodemographic, Army career, and mental health characteristics with suicide ideation among Regular Army enlisted Special Operations Forces (SOF).[a]

	SOF Element				
	Operators		Support		
Characteristics		OR	(95% CI)	OR	(95% CI)
Sex					
Male		1.0	–	1.0	–
Female		3.9	(1.7–8.9)	1.2	(0.8–1.7)
	χ^2_1 =	10.3* (p = .001)		1.0 (p = .32)	
Age					
≤ 24 years		1.4	(0.8–2.4)	2.0	(1.5–2.6)
25 – 29 years		0.7	(0.4–1.1)	1.4	(1.0–1.8)
30 – 34 years		1.0	–	1.0	–
≥ 35 years		0.8	(0.5–1.2)	0.9	(0.6–1.3)
	χ^2_3 =	7.5* (p = .056)		45.3* (p < .0001)	
Race-ethnicity					
White non-Hispanic		1.0	–	1.0	–
Other[b]		1.1	(0.7–1.7)	0.7	(0.6–0.9)
	χ^2_1 =	0.1 (p = .77)		13.7* (p = .0002)	
Marital status					
Not currently married		1.6	(1.1–2.4)	1.1	(1.0–1.3)
Currently married		1.0	–	1.0	–
	χ^2_1 =	6.0* (p = .015)		2.0 (p = .16)	
Rank					
E1 – E4		1.0	–	1.0	–
E5 – E6		0.5	(0.3–0.8)	0.5	(0.4–0.6)
E7 – E8		0.3	(0.2–0.5)	0.2	(0.1–0.3)
	χ^2_2 =	19.1* (p < .0001)		101.4* (p < .0001)	
Deployment status					
Currently/previously deployed		1.0	–	1.0	–
Never deployed		1.3	(0.7–2.3)	1.5	(1.2–1.8)
	χ^2_1 =	0.7 (p = .39)		18.1* (p < .0001)	
Demoted in the past year					
Yes		11.5	(5.3–24.8)	5.3	(4.1–6.9)
No[c]		1.0	–	1.0	–
	χ^2_1 =	38.6* (p < .0001)		167.1* (p < .0001)	
SOF status					
Currently SOF		1.0	–	1.0	–
Not currently SOF		2.1	(1.4–3.1)	2.1	(1.7–2.4)
	χ^2_1 =	13.7* (p = 0.0002)		68.6* (p < .0001)	
Mental health diagnosis[d]					
Yes		6.3	(4.1–9.9)	5.7	(4.7–6.9)
No		1.0	–	1.0	–
	χ^2_1 =	65.6* (p < .0001)		320.8* (p < .0001)	

[a] This sample of Regular Army enlisted Special Operations Forces, which includes all person-months with a first suicide ideation (n = 97 Operator cases; n = 568 Support cases) and an equal-probability sample of controls person-months (unweighted n = 5,942 Operator controls; unweighted n = 11,665 Support controls) is a subset of all soldiers in the 2004–2012 Army STARRS Historical Administrative Data Study (HADS). Control person-months were assigned a weight of 137.9 to adjust for under-sampling.

[b] The "other" race-ethnicity category includes: American Indian/Alaska Native, Asian, Black, Hispanic, and Native Hawaiian or other Pacific Islander. For a complete list of the original database categories, see eTable 3.

[c] Includes those who have never been demoted as well as those who were demoted more than a year ago.

[d] Administratively documented mental health diagnosis

SOF = Special Operations Forces

*p < .05

© 2025 Naifeh JA et al. *JAMA Network Open*.

eTable 9. Multivariable associations of sociodemographic, Army career, and mental health characteristics with suicide ideation among Regular Army enlisted Special Operations Forces (SOF).[a]

		SOF Element				
		Operators		Support		SOF Element ´ Characteristic Interaction[b]
Characteristics		OR	(95% CI)	OR	(95% CI)	
Sex						
Male		1.0	–	1.0	–	
Female		1.4	(0.6–3.4)	1.1	(0.7–1.6)	
	χ^2_1 =	0.5 (*p* = 0.48)		0.2 (*p* = 0.70)		0.1 (*p* = 0.76)
Age						
≤ 24 years		0.9	(0.5–1.8)	1.4	(1.0–1.9)	
25 – 29 years		0.5	(0.3–0.9)	1.1	(0.8–1.5)	
30 – 34 years		1.0	–	1.0	–	
≥ 35 years		0.9	(0.5–1.5)	1.4	(0.9–2.1)	
	χ^2_3 =	6.4 (*p* = .092)		9.4* (*p* = .025)		7.5 (*p* = .058)
Race-ethnicity						
White non-Hispanic		1.0	–	1.0	–	
Other[c]		0.9	(0.6–1.5)	0.8	(0.6–0.9)	
	χ^2_1 =	0.1 (*p* = .73)		8.2* (*p* = .004)		1.4 (*p* = .24)
Marital status						
Not currently married		1.2	(0.8–1.9)	1.0	(0.8–1.2)	
Currently married		1.0	–	1.0	–	
	χ^2_1 =	0.9 (*p* = .34)		0.1 (*p* = .82)		0.1 (*p* = .79)
Rank						
E1 – E4		1.0	–	1.0	–	
E5 – E6		0.5	(0.3–0.9)	0.5	(0.4–0.6)	
E7 – E8		0.2	(0.1–0.5)	0.1	(0.1–0.2)	
	χ^2_2 =	14.9* (*p* = .001)		74.0* (*p* < .0001)		4.4 (*p* = .11)
Deployment status						
Currently/previously deployed		1.0	–	1.0	–	
Never deployed		1.1	(0.6–2.0)	1.3	(1.1–1.6)	
	χ^2_1 =	0.1 (*p* = 0.80)		8.9* (*p* = .003)		1.5 (*p* = .23)
Demoted in the past year						
Yes		3.6	(1.5–8.3)	2.3	(1.7–3.0)	
No[d]		1.0	–	1.0	–	
	χ^2_1 =	8.6* (*p* = .003)		37.1* (*p* < .0001)		0.7 (*p* = .39)
SOF status						
Currently SOF		1.0	–	1.0	–	
Not currently SOF		1.6	(1.0–2.3)	1.7	(1.4–2.0)	
	χ^2_1 =	4.6* (*p* = 0.032)		35.1* (*p* < .0001)		0.0 (*p* = .87)
Mental health diagnosis[e]						
Yes		7.0	(4.4–11.1)	6.8	(5.6–8.2)	
No		1.0	–	1.0	–	
	χ^2_1 =	69.2* (*p* < .0001)		359.4* (*p* < .0001)		0.3 (*p* = .57)

\[a] This sample of Regular Army enlisted Special Operations Forces, which includes all person-months with a first suicide ideation (n = 97 Operator cases; n = 568 Support cases) and an equal-probability sample of controls person-months (unweighted n = 5,942 Operator controls; unweighted n = 11,665 Support controls) is a subset of all soldiers in the 2004–2012 Army STARRS Historical Administrative Data Study (HADS). Control person-months were assigned a weight of 137.9 to adjust for under-sampling.

[b] Each two-way interaction was examined in the total sample of SOF (including both Operators and Support soldiers) using a multivariable logistic regression model that adjusted for the main effects SOF role (Operator vs. Support) and all other sociodemographic and Army career variables in the table.

[c] The "other" race-ethnicity category includes: American Indian/Alaska Native, Asian, Black, Hispanic, and Native Hawaiian or other Pacific Islander. For a complete list of the original database categories, see eTable 3.

[d] Includes those who have never been demoted as well as those who were demoted more than a year ago.

[e] Administratively documented mental health diagnosis

SOF = Special Operations Forces

*$p < .05$

© 2025 Naifeh JA et al. *JAMA Network Open*.

eReferences for eMethods

1. Kessler RC, Colpe LJ, Fullerton CS, et al. Design of the Army Study to Assess Risk and Resilience in Servicemembers (Army STARRS). *Int. J. Methods Psychiatr. Res.* 2013;22(4):267-275. http://www.ncbi.nlm.nih.gov/pubmed/24318217
2. Willett JB, Singer JD. Investigating onset, cessation, relapse, and recovery: Why you should, and how you can, use discrete-time survival analysis to examine event occurrence. *J. Consult. Clin. Psychol.* Dec 1993;61(6):952-965. http://www.ncbi.nlm.nih.gov/pubmed/8113496.
3. Singer JD, Willett JB. *Applied longitudinal data analysis: Modeling change and event occurrence.* New York, NY: Oxford University Press; 2003.
4. Schlesselman JJ. *Case-control studies: Design, conduct, analysis.* New York, NY: Oxford University Press; 1982.
5. Gahm GA, Reger MA, Kinn JT, Luxton DD, Skopp NA, Bush NE. Addressing the surveillance goal in the National Strategy for Suicide Prevention: The Department of Defense Suicide Event Report. *Am. J. Public Health.* 2012;102(Suppl 1):S24-S28. https://pubmed.ncbi.nlm.nih.gov/22390595/
6. Centers for Disease Control and Prevention. The International Classification of Diseases, ninth revision, Clinical Modification (ICD-9-CM). 2013; https://www.cdc.gov/nchs/icd/icd9cm.htm. Accessed June 10, 2021. https://www.cdc.gov/nchs/icd/icd9cm.htm
7. SAS Institute Inc. *SAS® 9.4 Software.* Cary, NC: SAS Institute Inc.; 2013.
8. Roalfe AK, Holder RL, Wilson S. Standardisation of rates using logistic regression: A comparison with the direct method. *BMC Health Serv. Res.* 2008;8:275.

© 2025 Naifeh JA et al. *JAMA Network Open*.
