# Supplementary material for: Suicidal Behavior in US Army Special Operations Forces
Source: JAMA Netw Open. 2025 Aug 15;8(8):e2527395. doi: 10.1001/jamanetworkopen.2025.27395 (PMC12357185; doi:10.1001/jamanetworkopen.2025.27395)
Supplement: Supplement 2. — Data Sharing Statement [file jamanetwopen-e2527395-s002.pdf]

Data Sharing Statement

Naifeh. Suicidal Behavior in US Army Special Operations Forces. *JAMA Netw Open*. Published August 15, 2025. doi:10.1001/jamanetworkopen.2025.27395

Data
Data available: No

Additional Information
Explanation for why data not available: The STARRS HADS data used in this paper are not available for public release. DoD clearance is required to access this data. However, access to the Army STARRS and STARRS-LS survey data and project data (not used here) can be requested through the Interuniversity Consortium for Political and Social Research (ICPSR) at the University of Michigan (https://www.icpsr.umich.edu/web/ICPSR/studies/35197). ICPSR can currently provide data from 3 Army STARRS surveys: the All Army Study (AAS), the New Soldier Study (NSS), and the Pre/Post Deployment Study (PPDS).
